# 109 Frequency of Visible Long Term Scarring in Split Thickness Donor Sites

**DOI:** 10.1093/jbcr/iraf019.109

**Published:** 2025-04-01

**Authors:** Sigrid Blome-Eberwein, Sakura Helm, Shaliz Aflatooni, Nicole Natarelli

**Affiliations:** Lehigh Valley Health Network; Lehigh Valley Health Network; Lehigh Valley Health Network; University of South Florida Morsani College of Medicine

## Abstract

**Introduction:**

Split thickness skin grafting (STSG) is the standard treatment for burn wounds without healing potential (deep 2nd and 3rd degree) and other large full-thickness skin wounds. Harvesting a split thickness skin graft creates a new wound, called the donor site. While attention has recently been spent on the healing and scarring process of burn wounds, little light has been shed on the long-term outcomes of donor site wounds. Most (small) studies focus on healing time as opposed to long term sequelae. This study is an IRB-approved, retrospective chart review of the long-term outcomes of donor sites to identify the factors correlated with donor site dyspigmentation and scarring.

**Methods:**

Patients in this study were admitted to our burn center between May 22, 2015 – April 14, 2024. The study population was extracted from the trauma burn registry, and then manually reviewed in EPIC by study team members. Patient information reviewed included demographics, patient skin type, total body surface area (TBSA) burned, operative details (including the donor site location, dressing, and thickness), healing time of the donor site, complications, discomfort, and donor site morbidity were extracted from their chart. Patients of all ages were included in the study. Skin type was determined using the Fitzpatrick Scale. Photographs and clinical notes from the patient’s follow-up visits were utilized to assess donor site morbidity and/or discomfort.

**Results:**

1014 patients treated with a STSG were entered into the study. 66.3% were male, 33.5% were female., 77.8% white, 6.3% Black and 11.1% Hispanic. The average age was 46 at the time of grafting. The average depth of graft taken was 0.01 inches, in 90.3% from the thigh. 32.9% had dyspigmentation of donor sites on their second follow up visit and 5.8% had hypertrophic scarring. 1.5% ultimately required treatment for their donor site scarring in our institution. The most highly correlated factors in dyspigmented donor sites were higher age and Fitzpatrick Skin Types 4-5. Donor sites with hypertrophic scarring were found to be most correlated with Fitzpatrick Skin Types 4-5.

**Conclusions:**

Donor sites can leave long-term scarring and/or dyspigmentation in a significant percentage of patients treated with a split thickness skin graft, especially in darker skin type patients and older patients. Reduction of donor sites and take from inconspicuous areas during the initial treatment of these patients should be attempted.

**Applicability of Research to Practice:**

immediate

**Funding for the Study:**

Institutional foundation funding

